# Immunohistochemical study of epiretinal membranes in patients with uveitis

**DOI:** 10.1007/s12348-012-0074-x

**Published:** 2012-04-25

**Authors:** Arsham Sheybani, George J. Harocopos, P. Kumar Rao

**Affiliations:** 1 Department of Ophthalmology and Visual Sciences, School of Medicine, Washington University in St. Louis, CB 8096, 660 S. Euclid, St. Louis, MO 63110 USA; 2 Department of Pathology and Immunology, School of Medicine, CB 8096, 660 S. Euclid, St. Louis, MO 63110 USA

**Keywords:** Epiretinal membrane, Uveitis, Immunohistochemistry, Surgery, Inflammation, Cytokines, Histology

## Abstract

**Background:**

The purpose of this study is to report two cases of idiopathic uveitis with secondary epiretinal membrane (ERM) formation in order to describe histologic and immunohistochemical features that may help distinguish uveitic from idiopathic ERMs.

**Methods:**

The study utilized a clinical case series and histopathological and immunohistochemical findings.

**Results:**

There was no identifiable etiology of inflammation in either case. Histology and immunohistochemistry demonstrated a mixture of abundant inflammatory cells, including lymphocytes, histiocytes, plasma cells, and occasional eosinophils, among a stromal matrix composed of glial elements and condensed vitreous, but no retinal pigment epithelium (RPE) was present. The relative proportions of the various inflammatory cell types were assessed with immunohistochemistry, and among the lymphocyte population, T cells predominated over B cells. In one of the cases, there was an abundance of histiocytes, consistent with granulomatous uveitis, which was later confirmed on histology of the enucleated globe.

**Conclusions:**

Idiopathic ERM formation is thought to be secondary to glial cell migration that may require some involvement of RPE cells. The absence of RPE and abundance of inflammatory cells may be used to identify ERMs as secondary to uveitis.

## Introduction

Epiretinal membranes (ERM) are fibrocellular proliferations over the internal limiting membrane that can lead to significant macular pathology when associated with contraction [1]. When these membranes contract, patients often complain of metamorphopsia and loss of visual acuity. Early histopathologic studies characterized ERMs from a variety of diseases; however, immunohistochemical studies were not performed [2, 3]. While there is little understanding as to how idiopathic ERMs form, there is even less information regarding the formation of ERMs in chronic uveitis. To better understand the formation of these membranes, we aim to characterize the immunohistochemistry of ERMs from two patients with chronic idiopathic uveitis.

## Case report 1

A 50-year-old woman was referred for intraocular inflammation in the right eye with decreased vision for 2 days. She had no past medical history, and on exam, her vision was 20/400 in the right eye and 20/40 in the left. Dilated fundoscopic examination revealed 1+ vitreous cell and inferior snowballs and vitreous debris with a chorioretinal scar in the right eye. Examination of the left eye revealed nuclear sclerotic cataract and was otherwise normal. She was started on azithromycin for the possibility of toxoplasmosis-related chorioretinitis, and a systemic work-up was initiated, which was negative for infectious, inflammatory, and vasculitic processes. Over the next 2 months, her vision improved to 20/150 with a combination of topical prednisolone acetate 1 % and empiric therapy for toxoplasmosis. However, the amount of inflammation remained largely unchanged, and she underwent vitreous biopsy with the hope of identifying a causative agent.

**Fig. 1:**
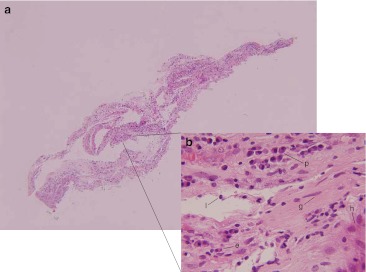
Histology of excised epiretinal membrane from case 1 (hematoxylin and eosin stain). **a** The membrane in its entirety, with abundant inflammatory cells (magnification, ×40). **b** High magnification (×600) shows an admixture of chronic inflammatory cells, including lymphocytes (*l*), plasma cells (*p*), histiocytes (*h*), and occasional eosinophils (*e*). Cells with spindly nuclei and surrounding fibroconnective tissue identify the fibro-glial component (*g*)

**Fig. 2:**
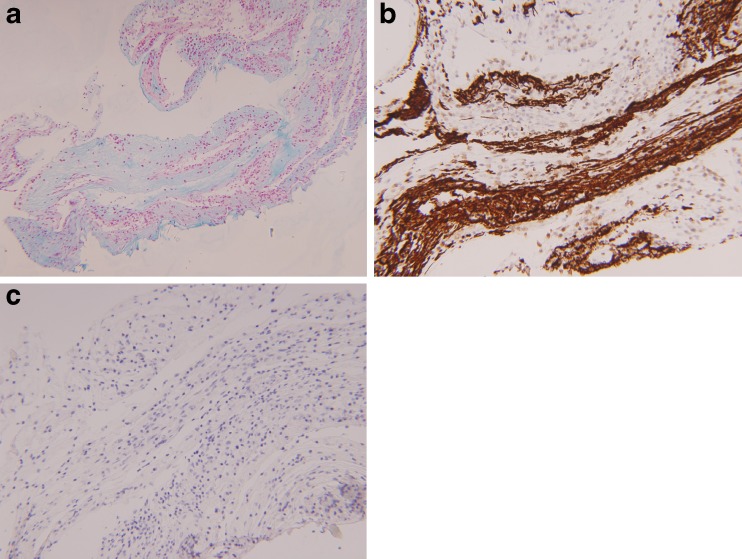
Special stains characterizing the stromal background of the epiretinal membrane from case 1. **a** Alcian blue stain, confirming the presence of acid mucopolysaccharide, consistent with condensed vitreous (magnification, ×100). **b** GFAP immunostain, highlighting the retinal glial elements (magnification, ×200). **c** CAM5.2-red immunostain is completely negative, confirming the absence of RPE (magnification, ×200)

**Fig. 3:**
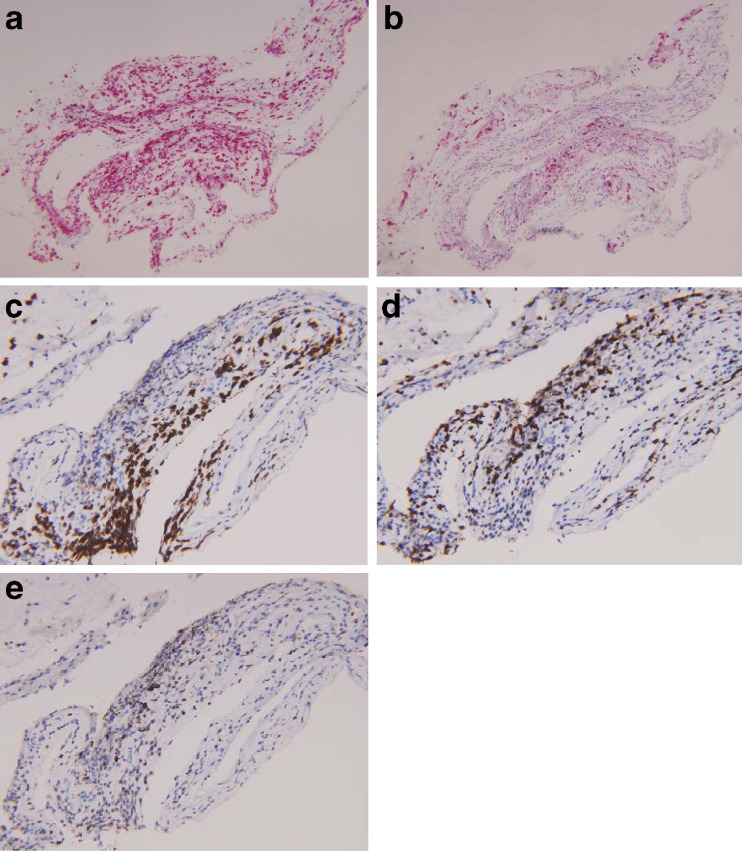
Immunohistochemical staining of the ERM from case 1, demonstrating a predominance of lymphocytes among the inflammatory cell population, and out of the lymphocyte population, a predominance of T cells over B cells. **a** CD45-red immunostain highlights lymphocytes (magnification, ×100). **b** CD68-red highlights histiocytes (magnification, ×100). **c** CD138 highlights plasma cells (magnification, ×200). **d** CD3 highlights T cells (magnification, ×200). **e** CD20 highlights B cells (magnification, ×200)

The biopsy results were consistent with intraocular inflammation without evidence of malignancy by cytological analysis, IgH gene rearrangement testing via PCR, flow cytometry, and cytokine concentration analysis. Cytologic smears showed a mixture of mature inflammatory cells. IgH gene rearrangement testing demonstrated polyclonality. Flow cytometry exhibited a preponderance of T cells over B cells (ratio of CD3-positive to CD19-positive cells = 72:1), and the CD19-positive population was too small to assess the kappa/lambda ratio. IL-6 from the vitreous fluid was measured at 239 pg/mL, and IL-10 at 18.2 pg/mL, yielding an IL-10 to IL-6 ratio more consistent with intraocular inflammation over malignancy [4]. No organisms were seen on gram stain and culture. At the time of surgery, it was also noted that the patient had a significant ERM in the macula, which was elevated, removed, and sent for histologic examination. Histology revealed abundant inflammatory cells, in addition to glial cells and condensed vitreous, without cells from the retinal pigment epithelium (RPE) (Figs. 1, 2, and 3). Immunohistochemical staining confirmed the membrane components: Retinal glia were highlighted by glial fibrillary acidic protein (GFAP), whereas CAM5.2 (cytokeratin 8/18) red was completely negative, verifying the absence of RPE (Fig. 2). Immunohistochemistry was also helpful for assessing the relative proportions of the various inflammatory cell types, including lymphocytes (CD45-red positive), histiocytes (CD68-red positive), and plasma cells (CD138 positive), with lymphocytes predominating. Out of the lymphocyte population, T cells (CD3 positive) predominated over B cells (CD20 positive), as would be expected in an inflammatory uveitis (Fig. 3).

## Case report 2

A 57-year-old woman with high myopia and a remote history of a prior retinal tear in the left eye treated with focal laser photocoagulation underwent pneumatic retinopexy and cryotherapy for a retinal tear in the right eye. She was referred for sudden onset of decreased vision 32 days after the recent procedure in the right eye. Her past medical history was significant for osteoarthritis and breast cancer treated with bilateral mastectomy 6 years prior to this presentation. Her vision had dropped from 20/25 to count fingers at 2 ft. Slit lamp examination revealed 2+ anterior chamber and vitreous cells without hypopyon. The retina was attached in all four quadrants, and the chorioretinal scarring from the cryotherapy procedure was seen inferotemporally. She was started on topical steroid therapy with prednisolone acetate 1 % with improvement in the inflammation, and her vision improved to 20/50 after 4 weeks of topical therapy. The steroid drop was slowly tapered; however, after 2 weeks, the inflammation worsened, and her vision declined to 2/200. A systemic work-up was initiated, and her topical steroid regimen increased. She returned 4 weeks later with no inflammation and 20/30 vision. Her systemic work-up was negative for infectious, inflammatory, and vasculitic processes known to cause intermediate and pan-uveitis. Her steroid drop was tapered, but 2 months later, she returned with 2+ cell and flare in the anterior chamber and vitreous cell, again without hypopyon. It was then decided to perform a vitreous biopsy to further investigate the cause of her recurrent inflammatory condition.

**Fig. 4:**
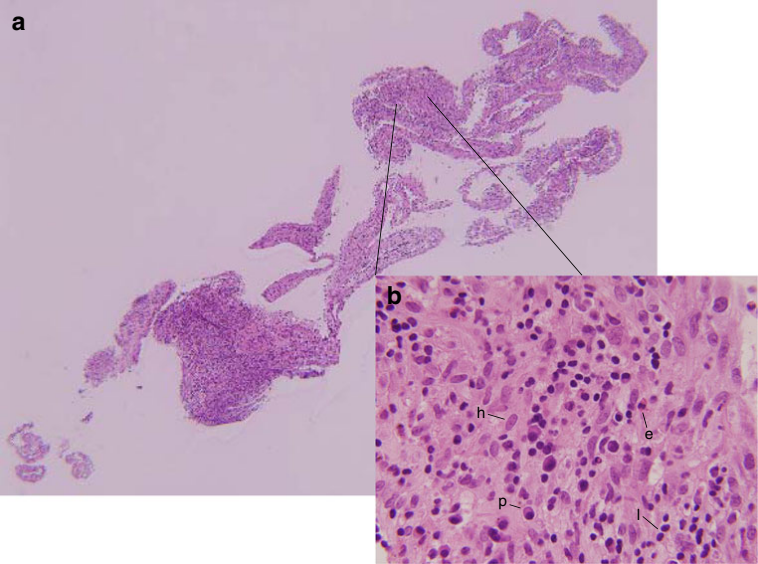
Histology of excised epiretinal membrane from case 2 (hematoxylin and eosin stain). **a** The membrane in its entirety, with abundant inflammatory cells (magnification, ×40). **b** High magnification (×600) shows an admixture of chronic inflammatory cells, including histiocytes (*h*), lymphocytes (*l*), plasma cells (*p*), and occasional eosinophils (*e*), with histiocytes predominating

**Fig. 5:**
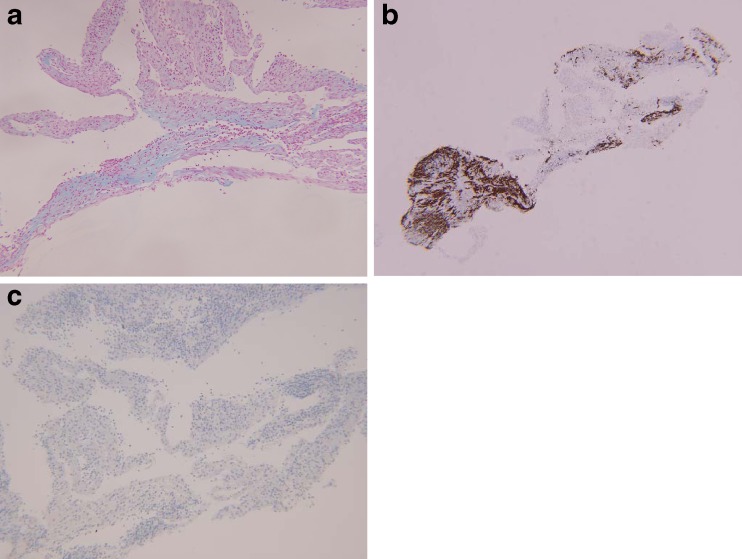
Special stains characterizing the stromal background of the epiretinal membrane from case 2. **a** Alcian blue stain, confirming the presence of acid mucopolysaccharide, consistent with condensed vitreous (magnification, ×100). **b** GFAP immunostain, highlighting the retinal glial elements (magnification, ×40). **c** CAM5.2-red immunostain is completely negative, confirming the absence of RPE (magnification, ×100)

**Fig. 6:**
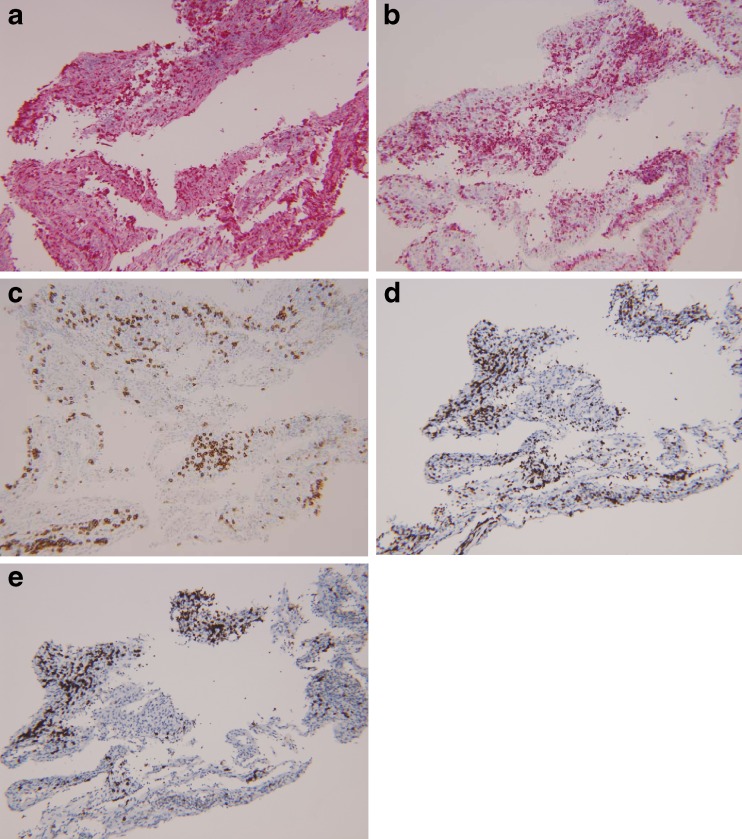
Immunohistochemical staining of the ERM from case 2, demonstrating a preponderance of histiocytes among the inflammatory cell population and out of the lymphocyte population, a slight preponderance of T cells over B cells (magnification, ×100). **a** CD68-red immunostain highlights lymphocytes. **b** CD45-red highlights lymphocytes. **c** CD138 highlights plasma cells. **d** CD3 highlights T cells. **e** CD20 highlights B cells

The biopsy results were consistent with intraocular inflammation without evidence of malignancy by cytological analysis, IgH gene rearrangement testing via PCR, flow cytometry, and cytokine concentration analysis. Cytologic smears showed a mixture of mature inflammatory cells, and polyclonality was found on IgH gene rearrangement testing. Flow cytometry demonstrated a preponderance of T cells over B cells (ratio of CD3-positive to CD19-positive cells = 69:1), and among the CD19-positive population, polyclonality was confirmed with a kappa/lambda ratio of 52:48. IL-6 from the vitreous fluid was measured at 5,184 pg/mL, and IL-10 at 3.3 pg/mL, yielding an IL-10 to IL-6 ratio more consistent with intraocular inflammation over malignancy [4]. No organisms were seen on gram stain and culture. At the time of surgery, it was also noted that the patient had a significant ERM which was removed and histologically consisted of a mixture of abundant inflammatory cells, in addition to glia and condensed vitreous, without cells from the RPE (Figs. 4, 5, and 6), as in the previous case. However, histiocytes were significantly more abundant than in the prior case, confirmed with immunostaining (CD68-red-positive cells), consistent with a granulomatous inflammatory process. T cells (CD3 positive) predominated over B cells (CD20 positive), but not by as wide a proportion as in the prior case (Fig. 6).

**Fig. 7:**
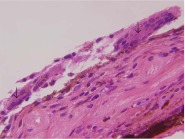
Histology of the enucleated globe from case 2. A granulomatous inflammatory membrane lines the iris surface, with two multinucleated giant cells (*arrows*) in the field. Chronic inflammatory cells, including histiocytes and lymphocytes, also infiltrate the iris stroma (hematoxylin and eosin stain; magnification, ×600)

Over the next 3 months, her clinical course deteriorated, as there was no resolution of a persistent anterior and intermediate uveitis. She eventually underwent retinal detachment repair with pars plana vitrectomy, air–fluid exchange, 360° of endolaser, and a 12 % C3F8 gas injection. Vitreous biopsy results were similar to her prior studies. Her clinical course progressed to recurrent retinal detachment, requiring repair with scleral buckling and silicone oil tamponade. Six months after this procedure, she was no longer able to perceive light, and given her chronic discomfort, she opted to undergo enucleation of the right eye. Histology of the enucleated globe showed a granulomatous inflammatory membrane, with giant cells, blanketing the angle structures and iris (Fig. 7) and forming a pupillary/cyclitic membrane, which was fused to a totally detached, gliotic retina. The granulomatous inflammatory membrane was intensely CD68-red positive (not shown). There was also a pocket of granulomatous vitritis in the small, residual vitreous cavity, all of which findings were consistent with a diagnosis of granulomatous anterior and intermediate uveitis.

## Discussion

Some of the early series describing the histology of ERMs were done over 30 years ago [4, 5]. The formation of a simple ERM is thought to be secondary to glial cell migration from the nerve fiber layer. GFAP expression in simple ERMs further demonstrates that the majority of these membranes derive from glial cells as originally described [3]. Early studies also suggested that RPE cells are generally necessary for ERM formation and likely accentuate ERM formation when in combination with glial cells [4]. These observations were confirmed in a recent study of internal limiting membranes from idiopathic macular holes [6]. While ERMs of different presumed etiologies were studied, characterization of the less common forms was minimally pursued.

Given the complexity of ERM formation, Snead et al. attempted to classify ERMs by histology and etiology, subdividing them into three categories: simple, tissue repair, and neovascular ERMs [7]. While not stated directly in the paper, uveitic ERMs would likely fall under the tissue repair category, in which the biological mediators are thought to involve the NF-κB pathway specifically driven by TGFβ, IL-6, and PDGF. Interestingly, IL-6 to IL-10 ratios from the vitreous biopsies in these two cases were extremely elevated, possibly suggesting some direct or indirect relationship between IL-6 levels and ERM formation in chronic uveitis. In vivo studies indeed suggest that IL-6 is an inducer of gliosis in the retina through changes in the transcription profile of glial cells [8].

The membranes in this series both lacked RPE cells but contained abundant inflammatory cells that distinguished them from simple ERMs. These features can help differentiate simple ERMs from uveitic ERMs. While glial cells are present in both, it is possible that the original observations by Kampik et al. were correct in that ERM formation requires some combination of glial, inflammatory, and/or RPE cell types. The lack of RPE and presence of inflammatory cells may be used to classify ERMs as secondary to uveitis when there is a question as to their etiology.
